# Silver Nanoparticle-Directed Mast Cell Degranulation Is Mediated through Calcium and PI3K Signaling Independent of the High Affinity IgE Receptor

**DOI:** 10.1371/journal.pone.0167366

**Published:** 2016-12-01

**Authors:** Nasser B. Alsaleh, Indushekhar Persaud, Jared M. Brown

**Affiliations:** Department of Pharmaceutical Sciences, Skaggs School of Pharmacy and Pharmaceutical Sciences, The University of Colorado Anschutz Medical Campus, Aurora, Colorado, United States of America; University of PECS Medical School, HUNGARY

## Abstract

Engineered nanomaterial (ENM)-mediated toxicity often involves triggering immune responses. Mast cells can regulate both innate and adaptive immune responses and are key effectors in allergic diseases and inflammation. Silver nanoparticles (AgNPs) are one of the most prevalent nanomaterials used in consumer products due to their antimicrobial properties. We have previously shown that AgNPs induce mast cell degranulation that was dependent on nanoparticle physicochemical properties. Furthermore, we identified a role for scavenger receptor B1 (SR-B1) in AgNP-mediated mast cell degranulation. However, it is completely unknown how SR-B1 mediates mast cell degranulation and the intracellular signaling pathways involved. In the current study, we hypothesized that SR-B1 interaction with AgNPs directs mast cell degranulation through activation of signal transduction pathways that culminate in an increase in intracellular calcium signal leading to mast cell degranulation. For these studies, we utilized bone marrow-derived mast cells (BMMC) isolated from C57Bl/6 mice and RBL-2H3 cells (rat basophilic leukemia cell line). Our data support our hypothesis and show that AgNP-directed mast cell degranulation involves activation of PI3K, PLCγ and an increase in intracellular calcium levels. Moreover, we found that influx of extracellular calcium is required for the cells to degranulate in response to AgNP exposure and is mediated at least partially via the CRAC channels. Taken together, our results provide new insights into AgNP-induced mast cell activation that are key for designing novel ENMs that are devoid of immune system activation.

## Introduction

The use of engineered nanomaterials (ENMs) in consumer and biomedical products is exponentially increasing and are being incorporated into a wide range of industries such as electronics, clothing, paints, detergents, cosmetics, biomedical imaging, drug delivery, etc. [1]. Advancements in nanotechnology and materials science have resulted in continuous introduction of novel ENMs into the market with a wide range of applications. It is now evident that exposure to ENMs is associated with toxicological adverse effects potentially due to their active surface area and wide disposition in different body tissues [2]. Over the past decade, much effort has been put into understanding physicochemical properties of ENMs and associated toxicities, that is, structure-activity relationship (SAR) of ENMs [3]. Nevertheless, little is known about ENM-associated toxicities at the cellular and molecular levels.

Silver nanoparticles (AgNPs) are one of the most utilized ENMs in consumer products largely due to their antimicrobial properties. AgNPs are incorporated into a variety of products including biomedical applications such as AgNP-coated medical devices and wound dressings [4]. Nevertheless, previous research provides evidence that exposure to AgNPs is associated with toxicological adverse effects in different organs including the lungs, kidneys and liver [5–8]. Furthermore, we and others have shown previously that AgNPs activate macrophages, through formation of reactive species to release a variety of inflammatory mediators, which can potentially lead to an activation of immune responses [9–11]. We recently demonstrated that some AgNPs, depending on their physicochemical properties, can activate mast cells [12]. Specifically, we found that spherical 20 nm but not 110 nm AgNPs (with two different particle coatings) induced mast cell degranulation dose-dependently suggesting that an inverse relationship between size of AgNPs and mast cell degranulation. Given the wide utilization of AgNPs in consumer products, assessment of immunomodulation and immunotoxicity of AgNPs is of crucial importance.

Mast cells are important effector cells that can regulate both innate and adaptive immune responses. They originate from the bone marrow (CD34^+^ pluripotent stem cells) and differentiate upon migration into tissues in the presence of necessary cytokines such as IL-3 and stem cell factor [13]. They are primarily located in areas with close contact to the external environment (e.g. mucosa, skin, etc.) and hence, they are considered first responders to pathogen invasion. Activation of mast cells can lead to an immediate release of preformed granules filled with mediators such as histamine, serotonin and proteases, which can recruit and activate a variety of immune cells [14]. Mast cells play a central role in allergy and inflammation, largely through the high-affinity IgE receptor type 1 (FcεR1). In addition to their role in allergic immune response, it was previously demonstrated that exposure to metals and transition metals, as components of particulate matter, led to mast cell activation and exacerbated allergen-mediated mast cell activation [15]. Therefore, it was reasonable to hypothesize that activation of mast cells in response to ENM exposure can potentially result in allergy-like symptoms. Indeed we have previously reported that a number of AgNPs with different physicochemical properties induced robust mast cell activation [12]. Accordingly, mast cells represent a good model for studying ENM-mediated immunomodulation and immunotoxicity as well as promotion of allergy-like responses.

Scavenger receptors (SR) comprise a large family that varies in regards to their ligand specificities and functions [16]. They were first identified for their role in recognizing oxidized form of lipoproteins [16]. Today we know that these receptors are involved in other essential biological functions such as recognition/removal of pathogens (and hence scavenger receptors are considered pattern recognition receptors, PRR). Some ENMs resemble the size and charge of pathogens and thus it was logical to hypothesize that scavenger receptors might be involved in ENM recognition and/or uptake. Indeed, we and others have previously shown that different classes of scavenger receptors are involved in recognition and uptake of a variety of nanoparticles [17–20]. Specifically, we identified scavenger receptor type B1 (SR-B1) to be involved in AgNP-directed mast cell degranulation [12].

One of the challenges for studying toxicity of ENMs is the continuous introduction and existence of a large number of ENMs with unique physicochemical properties. Over the past decade, numerous laboratories have studied SAR of ENMs in different in-vitro and in-vivo models. Although we have learned a great deal about the effect of different physicochemical properties of ENMs on biological systems, we are still unable to explain the behavior of many ENMs and we still lack knowledge about the underlying molecular mechanisms. We have previously shown that AgNPs induce mast cell degranulation through SR-B1 [12]; however, the underlying molecular mechanism is completely undetermined. Therefore, in this study, we sought to assess activation of intracellular signaling pathways in the mast cell response following exposure to AgNPs. Based on our previous studies, we utilized 20 nm AgNPs, which produced the most robust degranulation of mast cells. We hypothesized that AgNP-mediated interaction with SR-B1 involves activation of signal transduction pathways that culminate in increased intracellular calcium levels and degranulation of the mast cell. We have identified signaling components that are activated in response to AgNP exposure, thus providing new insights into cellular mechanisms of AgNP-mediated activation of mast cells.

## Materials and Methods

### Particle characterization

The hydrodynamic size and zeta potential (ZetaSizer Nano, Malvern) of AgNPs were characterized in DI water and N-2-hydroxyethylpip-erazine-N-2-ethane sulfonic acid (HEPES). At least 3 individual samples at a concentration of 25 μg/ml were used for measurements. Size and shape of AgNPs were confirmed by transmission electron microscopy (Tecnai Spirit Biotwin).

### Cell culture

RBL-2H3, a rat basophilic leukemia cell line, was obtained from the American Type Culture Collection (ATCC). Cells were cultured at 37°C and 5% CO_2_ in Eagle's Minimum Essential Medium (EMEM) media containing 15% heat-deactivated Fetal Bovine Serum (FBS) and 1.0% penicillin–streptomycin. Bone marrow-derived mast cells (BMMC) were isolated from femurs of male C57BI/6 mice (Jackson Laboratories, Bar Harbor, ME) at the age of 6–8 weeks. Cells were cultured for 4–6 weeks at 37°C and 5% CO_2_. BMMCs were cultured in RPMI 1640 medium supplemented with 10% FBS, 100 U/ml penicillin, 100 μg/ml streptomycin, 100 μg/ml Primocin^TM^ (Invitrogen, San Diego, CA), 25 mM HEPES, 1.0 mM sodium pyruvate, nonessential amino acids (BioSource International, Camarillo, CA), 0.0035% 2-mercaptoethanol and 300 ng/ml recombinant mouse IL-3 (PeproTech, Rocky Hill, NJ). Maturity and purity of BMMCs were assessed by measuring the expression level of FcεR1 and cKIT, respectively, as previously described [12]. All animal procedures were conducted in accordance with the National Institutes of Health guidelines and approved by the University of Colorado Denver Institutional Animal Care and Use Committee. Mice were maintained in individually ventilated, micro-isolated cages (4 mice per cage) under 12-hour light-dark cycles and fed ad libitum. Experiments were performed at least from 3 individual batches of mature mast cells. Each batch of mast cells was grown from femoral bone marrows of two mice.

### Reagents and antibodies

AgNPs with a diameter of 20 nm were procured from NanoComposix (San Diego, CA). For most studies, we used a concentration of 25 μg/ml based on our previous findings and others [12, 21]. AgNPs are suspended in citrate at a concentration of 1 mg/ml. Anti p-Tyr, p-Ser/Thr, p/t-plcγ1, p/t-PI3K were purchased from Cell Signaling (Beverly, MA), antibodies against SR-BI/II were purchased from LS BioSciences (Seattle, WA) and Thermoscientific (Waltham, MA). Cy^TM^5 Annexin V (BD Pharmingen) was purchased from BD Biosciences (San Jose, CA) and propidium iodide was purchased from Invitrogen (San Diego, CA). Inhibitors and reagents used in our studies include SR-B1 inhibitor 2-(2-butoxyethyl)-1-cyclopentanone thosemic-arbazone (Blt2) (Chembridge Corp., San Diego, CA, USA), Synta (compound 66) (Aobious INC, Gloucester, MA), N,N-Dimethylsphingosine (DMS), Wortmannin, U-73122, Ionomycin (Cayman Chem, Ann Arbor, MI), Ro 31–8220 (Abcam, Cambridge, MA). Concentrations of inhibitors were based on previous literature [22–26].

### Transmission electron microscopy

Cells were treated with 20 nm AgNPs (25 μg/ml) for 0, 10, 20, 30 or 60 min before they were high pressure frozen. Samples were vitrified by Wohlwend Compact O_2_ High Pressure Freezer and stored in 2% Osmium Tetraoxide/ 0.1% Uranyl Acetate/Acetone on dry ice overnight. Samples were gradually warmed up to -20°C for 24h, to 4°C for 4h, and then to room temperature for 1h. Samples were rinsed three times with EM Grade acetone and left in 25% Epson/75% acetone overnight. Infiltration was continued gradually to 50%E/50%A, 75%E/25%A and finally 100%Epson and left overnight. Accelerator was then added to Epson for 7h (rotator) after which samples were filled in beem capsules and centrifuged to bring the cells to the tip. Samples were polymerized in 60°C oven overnight. Blocks were removed from their mold (beem capsules) and Leica UC6 Ultramicrotome was used to make serial sections of 70nm. Finally, cells and particles were imaged using Tecnai Spirit Biotwin EM Microscope.

### Degranulation assay

Mast cell degranulation assay was carried out as previously described [12] based on the release of β-hexosaminidase. Briefly, BMMCs or RBL-2H3 were seeded at 5x10^4^ cells per well in 96-well flat-bottom plate. Cells treated later with dinitrophenylated human serum albumin (DNP-HSA) (100 ng/ml) (Sigma–Aldrich, St. Louis, MO) were sensitized with mouse anti-DNP IgE antibody (100 ng/ml) (Sigma–Aldrich, St. Louis, MO) 24h prior to treatment with DNP-HSA. For studies with inhibitors, cells were pre-treated with inhibitors 30 min prior to treatment with AgNPs. After 30 min incubation of DNP-HSA or 1h incubation of AgNPs (25 μg/ml), p-nitrophenyl-N-acetyl-b-D-glucopyranoside (PNAG) (Sigma–Aldrich, St. Louis, MO), a chromogenic substrate of β-hexosaminidase, was added to cell supernatants and lysates and incubated for 90 min at 37°C. The reaction was stopped with glycine (0.4M) and optical density was read at 405 nm using a Synergy^TM^ HT Multi-Mode Microplate Reader (BioTek Instruments Inc, Winooski, VT). We included nanoparticle-only controls to avoid any possible interference in the UV-Vis spectrum. Percentage of degranulation was calculated as follows: ((supernatant*2)/(lysate*4))*100 (using dilution factors). DMSO (vehicle) controls with final % of DMSO were included in these experiments to exclude any effect due to DMSO in inhibiting mast cell degranulation (% DMSO: 0.1% for Synta; 0.05% for Blt-2; less than 0.001% for other inhibitors).

### Intracellular calcium levels

Intracellular calcium levels were assessed using Fluo-4 AM (Molecular Probes, Life Technologies) according to the manufacturer's instructions. Briefly, Fluo-4 AM stock solution was prepared in DMSO at 1 mM. BMMCs were grown at 1*10^6^ cells/ml. Cells were centrifuged and pellets were gently re-suspended in 5 μM Fluo-4-containing supplement-free, phenol-free media for 20 min at 37°C protected from light. Following incubation, cells were washed 3 times and then were re-suspended in supplement-free, phenol-free media. Baseline calcium levels were recorded at 488 nm excitation/520 nm emission using Accuri^TM^ C6 flow cytometry (BD Biosciences, USA) (10,000 events), then cells were exposed to AgNPs for 2 min and mean fluorescence intensity was recorded. FCS Express 4 Software was used for data presentation (De Novo Software, Glendale, CA).

### Western blotting

RBL-2H3 or BMMCs were pretreated with indicated inhibitors and then exposed to AgNPs (25 μg/ml) for 5, 30 or 60 min. Cells were washed two times with ice-cold PBS and then ice-cold lysis buffer (80mM Tris HCl, 1% SDS, phosphatase inhibitors (Sigma), protease inhibitors (Sigma)) was added to cells and incubated for 45 min at 4°C. Cells were sonicated briefly and centrifuged at 10,000x *g* for 10min at 4°C. Protein content in the supernatants was measured using the Bradford reagent (Bio-Rad Laboratories Inc., Hercules, CA). 20 μg of protein was boiled for 5 min with 5% 2-mercaptoetahanol-containing 2x Laemmli Sample Buffer (Bio-Rad Laboratories Inc., Hercules, CA). Samples were ran in 12% SDS-polyacrylamide gels (Invitrogen, San Diego, CA) and transferred electrophoretically to nitrocellulose membranes which was then blocked with 5% BSA in TBS-T for 1 h at 4°C. Primary antibodies were incubated overnight, whereas horseradish peroxidase-linked-secondary antibodies were incubated for 1 h at room temperature. Membranes were developed using Pierce Chemiluminescent Substrate (Thermoscientific, Waltham, MA). Relative densitometry (of phospho- to total-protein then to the corresponding loading control) was normalized to non-treated control using Image J software (NIH, Bethesda, MD, USA).

### Cell viability

Cell viability was assessed using 3 - (4,5—dimethylthiazol—2—yl)- 5- (3- carboxmethoxyphenyl) - 2- (4—sulfophenyl) - 2H - tetrazolium (MTS) assay according to the manufacturer’s instructions (Promega, Madison, WI) as previously described [27]. Briefly, RBL-2H3 cells were grown to 90% confluency in 96-well plates (Costar). Cells were treated with AgNPs in a serum-free DMEM/F12 HyClone GE media (Logan, Utah) for 1 h. Cells were centrifuged to bring nanoparticles to the bottom of wells and supernatants were transferred to new plates and read at 490 nm (BioTek Synergy HT, BioTek, Winooski, VT). We included nanoparticle-only controls for any possible interference in the UV-Vis spectrum that may have occurred from nanoparticles which remained in supernatants.

Mature BMMCs were plated at 1x10^6^ cells/ml. Cells were washed twice with PBS and re-suspended in 3 μM propidium iodide-containing staining buffer at a ratio of 1*10^5^ and then 5 μl of Annexin V reagent (BD Biosciences) was added per sample and incubated at room temperature protected from light for 15 min. 10,000 events were measured using an Accuri^TM^ C6 flow cytometry (BD Biosciences, USA). FCS Express 4 Software was used for data presentation (De Novo Software, Glendale, CA).

### Statistical analysis

All data are presented as mean ± SEM and were analyzed by one-way ANOVA, with differences between groups assessed using Bonferroni post hoc tests. Graphs and analysis were presented using GraphPad Prism 5 software (GraphPad, San Diego, CA). Differences between two groups were analyzed by student’s *t*-test. Differences were considered statistically significant at p<0.05.

## Results

### AgNP characterization and cellular uptake

**Fig 1:**
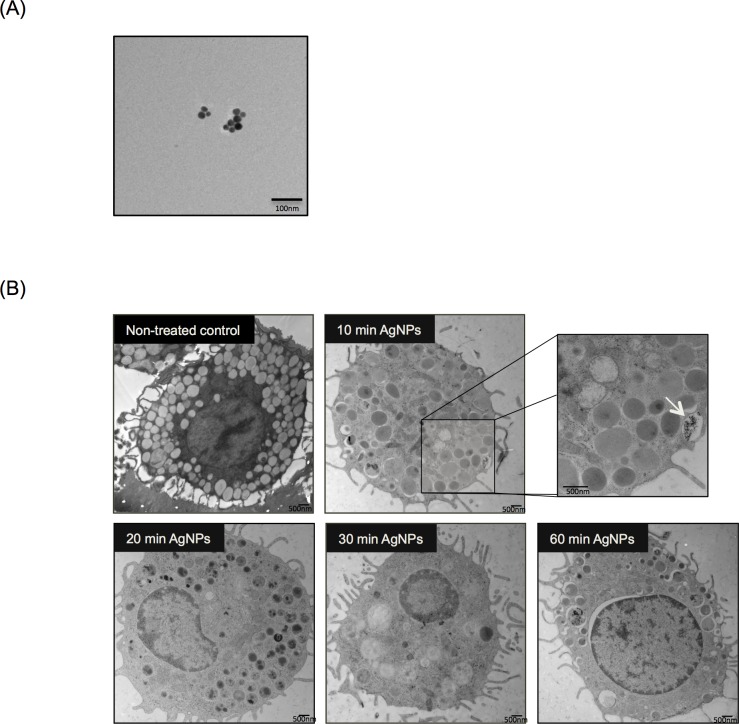
Time Course of Mast Cell Association of AgNPs (A) Representative Transmission Electron Microcopy (TEM) image demonstrating AgNP shape and size. (B) Representative TEM images of mast cells following exposure to 20 nm AgNPs over time. Mast cells were treated with AgNPs (25 μg/ml) for 10, 20, 30 and 60 min and AgNPs uptake by mast cells was assessed. Arrow indicates AgNPs that were being taken up by a mast cell (inset). A representative image was obtained from at least 5 different images.

AgNPs were characterized for their size, shape and charge. Hydrodynamic size and zeta potential were measured by dynamic light scattering (DLS) in water and HEPES buffer (which was used for the degranulation studies) (Table 1). Nanoparticles have a very large surface-to-volume ratio and therefore a change in their surface charge can significantly affect their behavior in aqueous solutions. It was previously demonstrated that a number of factors such as solution ionic strength, pH, surface charge, and surface coating influence particle stability consequently leading to particle aggregation and/or agglomeration [28]. The citrate-coated AgNPs utilized in our studies are stable in DI water (vehicle), as particle surface charge remains largely negative (~ - 40 mV) and thus no major aggregates were formed. However, it was found that as soon as AgNPs came in contact with the HEPES buffer, they formed aggregates (with a hydrodynamic size of ~150 nm and their zeta potential values decreased by ~ 10 mV) potentially as a result of interaction with the salts present in HEPES. This was indeed previously reported in the literature where gold nanoparticles showed agglomeration in the presence of salts [29]. The shape and size of AgNPs were also confirmed by transmission electron microscopy (TEM) (Fig 1A).

**Table 1:** Characterization of 20nm citrate-coated AgNPs in water and HEPES buffer

Water		HEPES	
Hydrodynamic Size (nm)	Zeta Potential (mV)	Hydrodynamic Size (nm)	Zeta Potential (mV)
29.71 ± 0.26	-39.10 ± 0.59	147.63 ± 13.29	-27.08 ± 1.21

We have previously shown (by utilizing inductively coupled plasma mass spectrometry, ICP-MS) that minimal uptake of 20 nm citrate-coated AgNPs occurs by mast cells within 1 hr [12]. In the current studies, our TEM results (Fig 1B) confirmed that only a very minimal amount of AgNPs were taken up by mast cells over a period of 1h exposure (inset Fig 1B). Following exposure to AgNPs, and as quickly as 10 min, mast cells started to degranulate (Fig 1B) as evidenced by membrane blebbing and reduction in the number of granules present in the cells over time. It was previously shown that exposure to silver ions (Ag^+^) induces mast cell apoptosis through cardiolipin oxidation and ATP depletion [30]. However, we assessed cellular toxicity in response to AgNPs (25 μg/ml) and found that 24 h exposure did not trigger any significant cell death (S1 Fig) suggesting a possible different mechanism between Ag^+^ and AgNPs in mediating mast cell activation/toxicity. Taken together, these data suggest that 20 nm AgNPs interact with the mast cell membrane leading to degranulation without induction of cellular toxicity.

### AgNP-mediated degranulation of mast cells is IgE independent and is distinct from classical allergen-mediated degranulation

**Fig 2:**
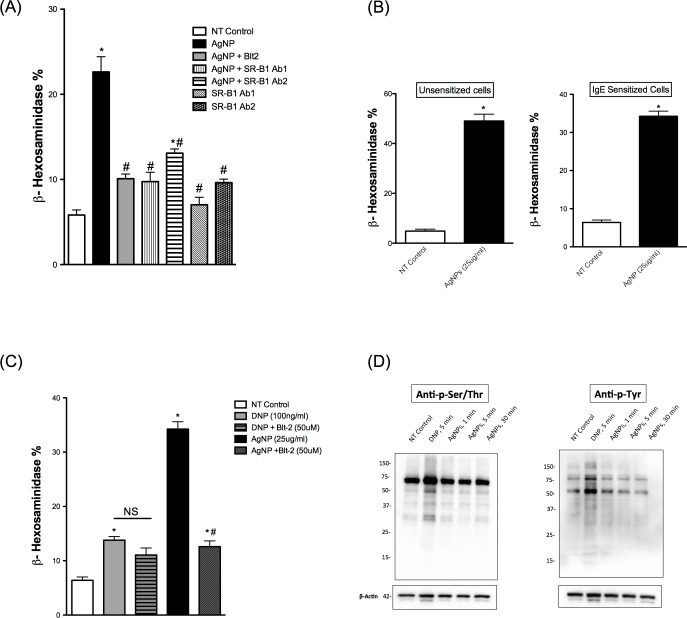
Mast cell degranulation following exposure to AgNPs Mast cell degranulation was assessed by measuring the release of β-hexosaminidase into supernatants. (A) Cells were pre-treated with the SR-B1 inhibitor Blt-2 (50 μM), SR-B1 specific antibodies (1:100 dilution) 30 min prior to AgNP (25 μg/ml) exposure for 1h and release of β-hexosaminidase was assessed. (B) Cells were sensitized with anti-DNP IgE overnight and then exposed to AgNPs (25 μg/ml) for 1 h and release of β-hexosaminidase was assessed. (C) Cells were pre-treated with Blt-2 (50 μM) for 30 min then activated with either DNP (30 min) or AgNP (1 h) and release of β-hexosaminidase was assessed. (D) Representative immunoblots for global p-Tyr and p-Ser/Thr following DNP (100 ng/ml) or AgNPs (25 μg/ml) exposure. Values are expressed as mean ± SEM of at least 3 independent experiments. *Indicates significant difference from controlled group (p≤0.05). #Indicates significant difference from AgNP-treated group (p≤0.05)

We have shown previously that Blt-2, a selective inhibitor of SR-B1, is able to inhibit mast cell degranulation in response to 20 nm AgNPs to an extent comparable to non-treated control [12]. Herein, we first confirmed the expression of SR-B1 in BMMCs (S3 Fig). We used specific antibodies against SR-B1 to confirm the inhibitor results. We found that two different polyclonal antibodies were able to block AgNP-induced degranulation of mast cells (Fig 2A) confirming the role of SR-B1 in AgNP-mediated activation of mast cells.

We have previously shown that mast cell degranulation in response to AgNPs does not require cell sensitization with IgE [12]. Here, we sought to assess mast cell degranulation in response to AgNPs following cell sensitization with anti-DNP IgE to determine whether FcεRI promotes AgNP-mediated degranulation. We found that robust mast cell degranulation occurred regardless of IgE sensitization (Fig 2B) suggesting that AgNP-induced mast cell degranulation does not involve FcεR1 activation. In order to test the possibility that the scavenger receptor inhibitor Blt-2 could block FcεRI-mediated degranulation of mast cells, we pre-treated cells with Blt-2 before treating them with DNP. Our results demonstrated that pre-treatment with Blt-2 did not block DNP-mediated mast cell degranulation (Fig 2C) suggesting a different mechanism of action from AgNP-induced mast cell degranulation. In addition, we assessed the signal transduction pathways following exposure to AgNP versus FcεRI-mediated degranulation using global p-tyrosine and p-serine/threonine. Probing whole cell lysates of cells exposed to AgNPs or DNP against anti- p-tyrosine and anti- p-serine/threonine suggests that DNP mediates a rapid phosphorylation of a large number of proteins, unlike AgNPs where there appears to be fewer phosphorylated proteins. Some of these appeared to overlap between DNP and AgNPs, for the most part (e.g. p-Tyr blot at ~55 kDa), and a few others appeared to be DNP specific (e.g. p-Ser/Thr at ~45 kDa) (Fig 2D). Based on our individual protein analysis (subsequent sections), separating proteins based on size and isoelectric point (using 2D gels) would possibly give a better resolution in highlighting differences between AgNP-exposed and NT samples. Taken together, our results suggest that AgNP exposure leads to activation of signal transduction pathways through a mechanism that is distinct from FcεRI-mediated mast cell degranulation further indicating a potential non-FcεR1 AgNP-mediated activation of mast cells.

### AgNP-mediated degranulation of mast cells requires influx of extracellular calcium partially through the CRAC calcium channels

**Fig 3:**
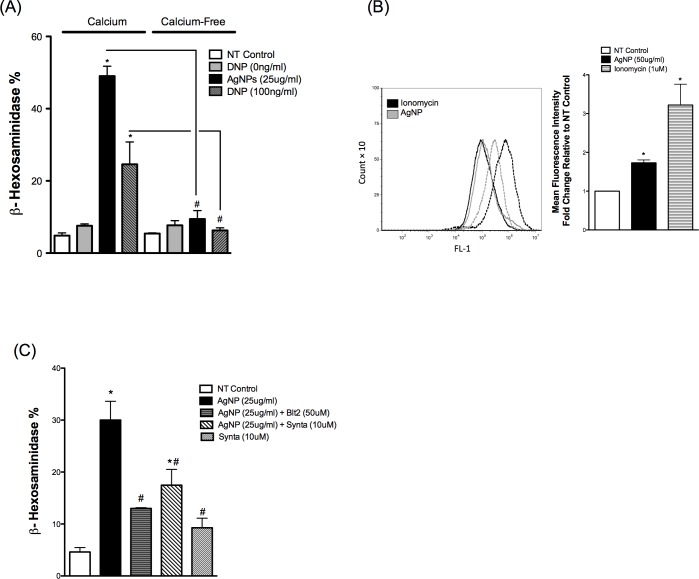
Calcium signal in mast cell following exposure to AgNPs Mast cell degranulation was assessed by measuring the release of β-hexosaminidase into supernatants. (A) Mast cells degranulation was measured following exposure to AgNPs in the presence and absence of calcium. (B–left panel) Cells were stained with Fluo-4 AM (5 μM) and mean fluorescence intensity was assessed before (baseline NT control, solid line) and after exposure to ionomycin (1 μM) or AgNPs (50 μg/ml) (dotted line) for 2 min. (B–right panel) A representative graph of 3 independent experiments showing fold change of mean fluorescence intensity relative to NT control. (C) Cells were pre-treated with the CRAC calcium channels inhibitor Synta (10 μM) 30 min prior to AgNP (25 μg/ml) exposure for 1 h and release of β-hexosaminidase was assessed. Values are expressed as mean ± SEM of at least 3 independent experiments. *Indicates significant difference from controlled group (p≤0.05). #Indicates significant difference from indicated groups (p≤0.05)

Because intracellular (cytosolic) calcium concentration [Ca^2+^]_i_ levels represent an important component of the signaling during mast cell activation and degranulation, it was vital to assess [Ca^2+^]_i_ following exposure to AgNPs. It was shown previously that mast cell degranulation requires influx of extracellular calcium in response to different IgE and non-IgE stimuli (e.g. stem cell factor, GPCR ligands, C3a) [31]. Accordingly, we first assessed the requirement of extracellular calcium influx in mast cell degranulation following exposure to AgNPs by measuring mast cell degranulation in the presence or absence of calcium in cell culture media. Our results demonstrate that mast cell degranulation was not increased following AgNP exposure in the absence of extracellular calcium (Fig 3A). Similarly and as previously reported in the literature [31], the presence of extracellular calcium was required for FcεRI-mediated mast cell degranulation (Fig 3A). These data suggest that AgNP-mediated degranulation involves an influx of extracellular calcium which subsequently led to an increase in [Ca^2+^]_i._ and degranulation of the cells. To confirm these results, we assessed the intracellular calcium signal following AgNP exposure. We have included ionomycin (an ionophore commonly used to induce mast cell degranulation) as a reference and positive control. Ionomycin, which was previously shown to induce a rapid and robust mast cell degranulation (about 70–80% degranulation) [32], resulted in about a three-fold increase in mean fluorescence intensity relatively to baseline (Fig 3B). Similarly, exposure to AgNPs induced almost a two-fold increase in mean fluorescence intensity (Fig 3B) confirming the requirement and influx of extracellular calcium in response to AgNP exposure. We next determined whether influx of extracellular calcium in response to AgNPs is mediated through cell membrane calcium channels or simply leakage into cells due to cell membrane damage. We found that Synta (10 μM), a selective inhibitor of the calcium release-activated channels (CRAC), was able to partially inhibit AgNP-mediated degranulation of mast cells (Fig 3C). Synta by itself induced an increase in mast cell degranulation (Fig 3C), which might be indicative of cell toxicity. We assessed cell death following exposure to Synta (10 μM) by Annexin V/PI staining. Exposure to Synta (10 μM) for 1.5 h did not induce any significant cytotoxicity compared to non-treated cells (data are not shown). Similarly, the cytotoxicity of Synta was previously assessed in CD8 cells and it was found that exposure to Synta (up to 10 μM) for 1 h did not result in increased cytotoxicity [33]. Taken together, these results suggest the important role of calcium in response to AgNP-mediated degranulation of mast cells

### AgNP-mediated mast cell degranulation involves activation of PLCγ and PI3K

**Fig 4:**
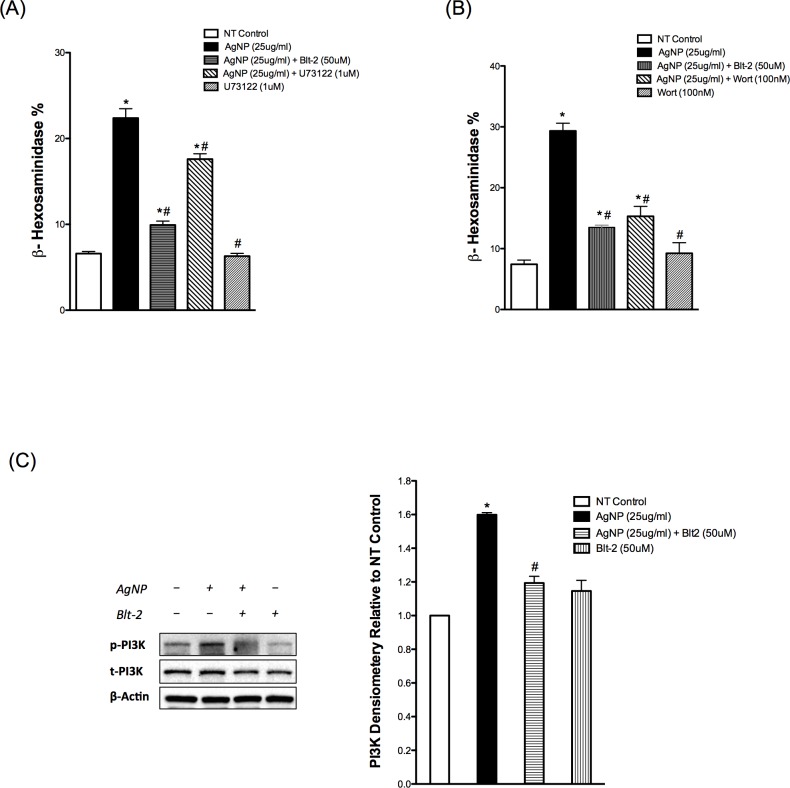
PLC and PI3K signaling in response to AgNPs Mast cell degranulation was assessed by measuring the release of β-hexosaminidase into supernatants. Cells were pre-treated with (A) the PLCγ inhibitor U73122 (1 μM) or (B) the PI3K inhibitor wortmannin (100 nM) 30 min prior to AgNP (25 μg/ml) exposure for 1 h and release of β-hexosaminidase was assessed. (C) Representative immunoblots for p-PI3K in samples pretreated with or without Blt-2 (50 μM) and followed by AgNP exposure for 1 h. Values are expressed as mean ± SEM of at least 3 independent experiments. *Indicates significant difference from controlled group (p≤0.05). #Indicates significant difference from AgNP-treated group (p≤0.05)

After we demonstrated the importance of the calcium signal in mast cell degranulation in response to AgNP exposure, we sought to assess major upstream signaling molecules that regulate the calcium signal in mast cells. One major signaling molecule is phospholipase Cγ (PLCγ), which utilizes the conversion of phosphatidyl inositol bisphosphate (PIP_2_) into two second messengers, inositol triphosphate (IP_3_) and diacylglycerol (DAG) [31, 34]. Binding of IP_3_ to its receptor in the endoplasmic reticulum (ER) induces the release of calcium from ER stores and depletion of ER calcium stores stimulates the influx of extracellular calcium, a phenomenon known as store-operated calcium entry (SOCE) [31, 34]. To assess the role of PLCγ in AgNP-mediated alteration in [Ca^2+^]_i_, we used a specific, commonly utilized inhibitor, U-73122. We demonstrated that mast cell degranulation in response to AgNPs was inhibited in cells pre-treated with U-73122 (Fig 4A). However, it was a partial inhibition, which possibly suggests the involvement of other pathways in AgNP-directed mast cell degranulation.

Another major upstream signaling molecule to PLCγ is phosphoinositide 3-kinase (PI3K), which has been shown to regulate the calcium signal in mast cells [35]. Furthermore, PI3K represents a pivotal signaling molecule for many cell surface receptors by regulating various signal transduction pathways involved in cell proliferation, differentiation, gene expression and cytokine release [36]. Here, we utilized the widely used irreversible noncompetitive inhibitor, wortmannin. Our results show that pre-treatment with wortmannin almost completely inhibited mast cell degranulation following AgNP exposure (Fig 4B) suggesting that PI3K is an upstream kinase that regulates PLCγ and other potential signaling pathways that are involved in AgNP-directed mast cell degranulation. In order to confirm these results, we assessed the phosphorylation status of PI3K following AgNP exposure. We found that expression of phosphorylated PI3K increased in AgNP-exposed cells thereby confirming the role of PI3K in AgNP-mediated mast cell degranulation (Fig 4C). In addition, pre-treatment with the SR-B1 antagonist Blt-2 abolished AgNP-mediated phosphorylation of PI3K (Fig 4C). Taken together, our studies suggest that AgNPs activate signal transduction pathway(s), putatively through SR-BI, that involves activation of PI3K and PLCγ and subsequent release of the second messengers, DAG and IP_3_, culminating in influx of extracellular calcium and degranulation of mast cells.

### PKC, but not sphingosine signaling, contributes to AgNP-directed mast cell degranulation

**Fig 5:**
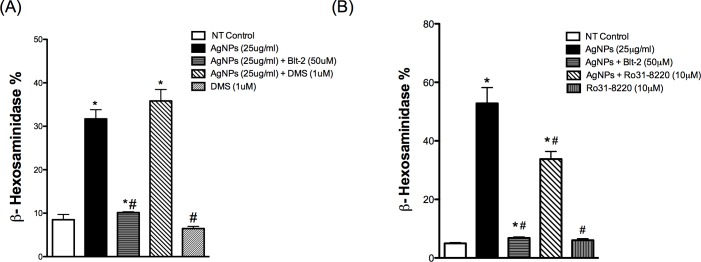
Involvement of other signaling pathways in response to AgNPs Mast cell degranulation was assessed by measuring the release of β-hexosaminidase into supernatants. Cells were pre-treated with (A) the sphingosine kinase inhibitor DMS (1 μM) or (B) the PKC inhibitor Ro31-8220 (10 μM) for 30 min prior to AgNP (25 μg/ml) exposure for 1 h and release of β-hexosaminidase was assessed. Values are expressed as mean ± SEM of at least 3 independent experiments. *Indicates significant difference from controlled group (p≤0.05). #Indicates significant difference from AgNP-treated group (p≤0.05)

In order to have a better understanding of the mechanism of AgNP-induced activation of signal transduction pathway(s) and degranulation of the mast cell, we sought to assess the contribution of other signaling molecules that have previously been shown to play a role in mast cell degranulation in response to antigen stimulation [31]. It has been shown that activation of PI3K leads to phospholipase D (PLD) activation, which in turn phosphorylates and activates sphingosine kinases (SphK) 1/2 that catalyzes the conversion of sphingosine to sphingosine-1-phosphate (S1P), a lipid signaling molecule. S1P diffuses out of the cell and interacts with its receptor and eventually this leads to the formation of IP_3_ and elevation of intracellular calcium levels [Ca^2+^]_i_ [31]. Further, S1P has also been shown to elevate [Ca^2+^]_i_ through an IP_3_-independent mechanism [31]. Blocking SphK with the competitive inhibitor N,N-Dimethylsphingosine (DMS) did not result in inhibition of AgNP-induced mast cell degranulation (Fig 5A) suggesting that mast cell degranulation in response to AgNP exposure does not involve formation/activation of the S1P pathway.

Another important signaling molecule that can contribute to mast cell degranulation is protein kinase C (PKC) [37]. PKC is a downstream signaling molecule to PLCγ, which is phosphorylated and activated by the action of the second messenger DAG. We utilized the selective inhibitor of PKC, Ro 31–8220. Mast cells have been reported to express PKCα, β, γ, δ, ε, η, θ, and ξ [38], while Ro 31–8220 inhibits the conventional isoforms of PKC (α, β and γ) and to lower extents atypical PKC isoforms (ε and ζ) [39–41]. We found that AgNP-mediated degranulation of mast cells was partially inhibited with Ro 31–8220 (Fig 5B) suggesting PKC contribution to mast cell degranulation in response to AgNPs.

### Confirmation of key signaling pathways in another mast cell model, RBL-2H3 cells

**Fig 6:**
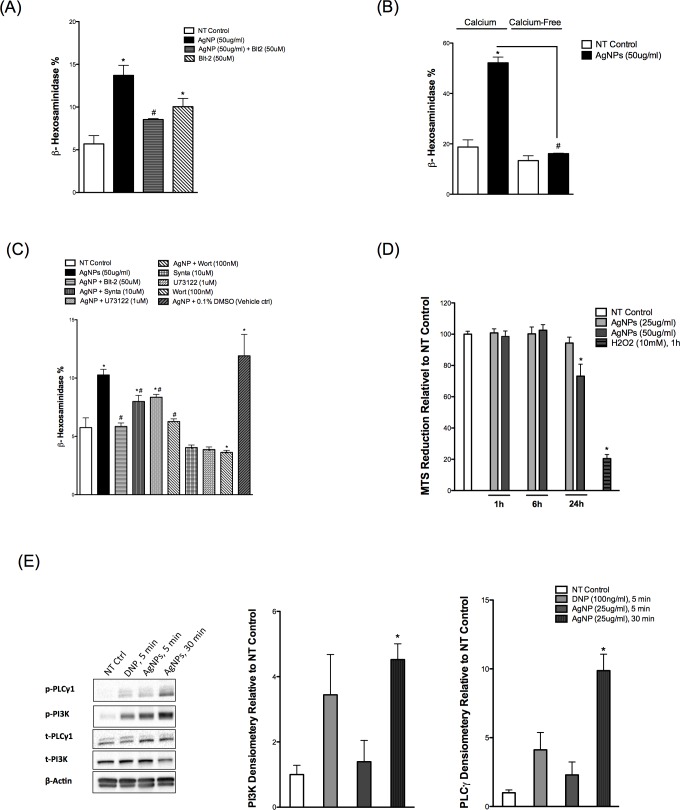
Confirmation of BMMCs results in RBL-2H3 (A) Cells were pre-treated with Blt-2 (50 μM) 30 min prior to AgNP (50 μg/ml) exposure for 1 h and release of β-hexosaminidase was assessed into supernatants. (B) Mast cells degranulation was measured following exposure to AgNPs (50 μg/ml) in the presence and absence of calcium. (C) Cells were pre-treated with the indicated inhibitors 30 min prior to AgNP (50 μg/ml) exposure for 1 h and release of β-hexosaminidase was assessed into supernatants. (D) Cells were treated with AgNPs (25 or 50 μg/ml) for 1, 6, and 24 h and cell viability was assessed by measuring the conversion of MTS into formazan. (E) Representative immunoblots for p-PLCγ and p-PI3K of mast cell in the presence or absence of DNP (100 ng/ml) for 5 min or AgNPs (25 μg/ml) for 5 and 30 min. Values are expressed as mean ± SEM of at least 3 independent experiments. *Indicates significant difference from controlled group (p≤0.05). #Indicates significant difference from AgNP-treated group (p≤0.05)

In order to confirm and validate our recent findings in another cell model, we obtained RBL-2H3, a rat basophilic leukemia cell line that has been extensively used in mast cell signaling studies. We confirmed the role of SR-B1 in inhibiting AgNP-mediated degranulation of these cells through use of Blt-2 (Fig 6A). RBL-2H3 cells appear to be less sensitive to AgNP-mediated degranulation compared to BMMCs and thus, we utilized a higher concentration of AgNPs (i.e. 50 μg/ml) to produce a significant amount of degranulation. Blt-2 appears to induce minor degranulation of the cells by itself (Fig 6A), which might suggest a cell toxicity effect. We assessed Blt-2 toxicity in BMMCs (PI/Annexin staining) and RBL-2H3 cells (MTS assay); however, we did not observe significant toxicity (S2 Fig). We confirmed the requirement of extracellular calcium in AgNP-mediated degranulation of RBL-2H3 cells (Fig 6B). Then we assessed the inhibition of AgNP-mediated degranulation by the inhibitors we used previously with BMMCs. We found a similar trend of inhibition previously seen with BMMCs (Fig 6C). Next we assessed the metabolic activity of the RBL-2H3 cells to confirm that the higher dose is not associated with cell toxicity. We found that 50 μg/ml AgNPs did not cause any significant cytotoxicity within 6 hours following AgNP exposure (Fig 6D). However, 24h of 50 μg/ml AgNPs exposure resulted in cytotoxicity (Fig 6D). Lastly, we wanted to confirm PI3K phosphorylation following AgNP exposure. We found a similar trend here as well i.e. exposure to AgNPs resulted in a robust, time-dependent increase in phosphorylation of PI3K following AgNP exposure (Fig 6E). Furthermore, we assessed the phosphorylation status of PLCγ1 and found that PLCγ1 was phosphorylated in a time-dependent manner as well in response to AgNP exposure (Fig 6E). Taken together, our RBL-2H3 data confirmed our previous BMMCs results and support our hypothesis for activation of key signal transduction pathways in mast cells exposed to AgNPs without major cytotoxicity.

## Discussion

One of the major challenges in the field of nanotoxicology is the existence of almost unlimited number of ENMs with unique physicochemical properties, making it unfeasible to compare the vast majority of nanotoxicity studies. Despite that and along with consortium efforts [42], we have improved our understanding of structure activity relationships (SAR) of ENMs in various in-vitro and in-vivo models over the last recent years [3]. However, the underlying molecular mechanisms of toxicity are poorly understood and delineation of such mechanisms is warranted for better design of novel ENMs that are devoid of major toxicity. In the current study, we sought to understand the cellular mechanisms of AgNP-mediated mast cell activation. We have demonstrated for the first time that AgNP-directed mast cell degranulation involves interaction with SR-B1 and activation of cell signal transduction pathways, which culminates in influx of calcium (through CRAC calcium channels) to induce degranulation of mast cells.

Different mechanisms of ENM uptake have been described and generally a positive correlation exists between uptake and toxicity of ENMs [43]. However, ENMs have also been shown to interact with cell surface receptors and activate signal transduction pathways. For instance, it was previously demonstrated that ultrafine carbon particles and amorphous silica (14nm in diameter) induce cell proliferation through interaction with the cell surface receptors, epidermal growth factor receptor (EGFR) and β1-integrin, inducing their downstream signaling pathways [44]. Another study has also shown that superparamagnetic iron oxide nanoparticles activate EGFR and its downstream signaling molecules [45]. Other reports have implicated Toll-like receptors and their adaptor molecules in ENM-mediated activation of cells [46, 47]. These studies suggest that interaction of ENMs with cell surface receptors is a potential mechanism of ENM-mediated biological responses. Our previous studies demonstrated that uptake of AgNPs by mast cells was minimal following exposure to 20nm AgNPs, yet produced the most robust degranulation of mast cells suggesting that mast cell activation in response to AgNPs is potentially mediated through interaction with a cell membrane receptor [12]. Our current TEM studies confirmed that AgNP uptake by mast cells is indeed minimal, however, degranulation of mast cells occurred within minutes following exposure to AgNPs and the number of secretory granules was clearly decreasing over time as an indication of degranulation. This further supports our hypothesis that AgNP-mediated degranulation of mast cells occurs through a membrane interaction rather than cellular uptake and requires a minimal number of AgNPs to induce a significant response. It’s worth mentioning that previous reports showed that dissolution of silver ions (Ag^+^) from AgNPs is attributed to AgNP-mediated toxicity [48–51] including degranulation and activation of mast cells [52]. However, our previous studies demonstrated that exposing mast cells to Ag^+^ did not induce their degranulation indicating that the particulate form of AgNPs is required for mast cell degranulation [12]. In support of a differential biological response between AgNPs and Ag^+^, it was previously reported that AgNPs exert more toxicity to several strains of bacteria as well as to the ryegrass *Lolium multiflorum* compared to an equivalent concentration of Ag^+^ [53, 54]. One study demonstrated that toxicity of AgNPs in human hepatoma cells is primarily due to oxidative stress that was independent of the toxicity of Ag^+^ [55]. A recent study has shown that AgNPs, but not Ag^+^, resulted in significant uptake and dysfunction of endothelial cells [56]. Furthermore, a recent in-vivo study has found a distinct tissue distribution and toxicity of Ag^+^ compared to AgNPs following a single dose of intravenous injection [57]. These studies suggest a potential toxicity due to the particles rather than Ag^+^. Taken together, our findings suggest that 20 nm AgNPs induce mast cell degranulation through a membrane and/or receptor-mediated mechanism that is not Ag^+^ dependent.

Mast cells are classically activated through the IgE (FcεR1) pathway. Other, less characterized pathways of mast cell activation also exist and can mediate mast cell degranulation independently of the FcεR1 [31]. Our current studies indicate that AgNP-induced mast cell activation occurs independent of the classic FcεR1 pathway, however, it involves activation of common signaling molecules that are involved in the regulation of the calcium signal. This is evidenced by our study of global phosphorylation of proteins on tyrosine, serine and threonine-residues, which shows a very robust and quick phosphorylation of a large number of proteins in response to IgE-mediated activation (as reported previously [58]), versus these of AgNPs, and yet AgNPs resulted in a more robust mast cell degranulation compared to IgE-mediated degranulation [12]. Another observation that suggests a distinct mechanism of AgNP-mediated mast cell degranulation to that of IgE-mediated degranulation was that the selective inhibitor of SR-B1 did not inhibit mast cell degranulation in response to FcεR1 stimulation, but clearly blocks AgNP mediated degranulation. Overall, our current results support our hypothesis that AgNP-mediated mast cell degranulation is distinct from IgE-mediated degranulation and involves interaction with SR-B1.

Scavenger receptors are a large family of receptors with different classes and members that share low homology and recognize a vast range of ligands such as oxidized lipoproteins and microbial structures [16]. SR-B1, a receptor for high-density lipoprotein (HDL), mediates uptake of HDL-derived cholesteryl esters from periphery to the liver (referred to as *reverse cholesterol transport*) and therefore it plays a pivotal role in cholesterol homeostasis and pathophysiology of atherosclerosis [59]. Further, SR-B1 has been shown to be involved in recognition and uptake of pathogens [60]. We and others previously demonstrated that scavenger receptors mediate interaction and uptake of ENMs [17–20]. Moreover, our previous studies show that a selective inhibitor of SR-B1 was able to inhibit mast cell degranulation and macrophage activation following AgNPs exposure to an extent comparable to non-treated cells [9, 12]. It was previously demonstrated that anti-SR-B1 antibodies resulted in blockage of SR-B1-mediated biological responses [61]. Our results show that AgNP-mediated degranulation of mast cells was almost completely abolished in cells pre-treated with anti-SR-B1 antibodies thereby confirming our previous results [12] for the involvement of SR-B1 in AgNP-mediated activation of mast cells. Nevertheless, the mechanism of AgNP-directed mast cell degranulation, potentially through SR-B1, at the molecular level is completely unknown. It was previously demonstrated that signaling through SR-B1 involves activation of signal transduction pathways including mitogen-activated protein kinases (MAPK), PI3K and PKC [62, 63]. Furthermore, it was shown in B-cells that influx of calcium in response to oligodeoxynucleotides (CpG) treatment is SR-B1 dependent [64] suggesting that SR-B1-mediated signaling involves regulation of the calcium signal. It has been well established that degranulation of mast cells to IgE and non-IgE stimuli requires an increase in cytosolic calcium levels ([Ca^2+^]_i_) and influx of extracellular calcium [31]. Accordingly, in the current study, we investigated the role of the calcium signal and its upstream regulator molecules such as PI3K and PLCγ following exposure to AgNPs.

Calcium is a versatile second messenger that regulates numerous cellular functions such as proliferation, contraction and secretion [65]. Many membrane receptors (e.g. tyrosine-coupled receptors, G-protein coupled receptors, etc.) function by activating signaling pathways that involve activation of different isoforms of phospholipase C (PLC) and generation of IP_3_ that eventually leads to an increase in [Ca^2+^]_i_ [66]. Previous work has demonstrated that various ENMs can induce a sustained increase in [Ca^2+^]_i_ in different cell models including mast cells [67–69]. Our current studies show that exposing mast cells to AgNPs resulted in a rapid increase in [Ca^2+^]_i_. Furthermore, influx of extracellular calcium was required for AgNP-induced mast cell degranulation. Depletion of ER calcium stores stimulates the formation of calcium release-activated channels (CRAC) to replenish ER stores [31] resulting in a sustained increase in calcium levels. Several inhibitors have been shown to block CRAC channels such as SKF-96365 and Synta (compound 66, GSK1349571A), with the later lacking any effects on inwardly rectifying K^+^ channels or plasma membrane calcium ATPase pump at a concentration of 10 μM [25]. Our data show that pre-treatment with Synta resulted in only a partial inhibition of AgNP-induced mast cell degranulation suggesting that other calcium channels might be involved in mediating calcium influx in response to AgNP exposure such as vanilloid receptor–related transient receptor potential (TRPV) that has been previously shown to mediate calcium influx during mast cell degranulation [70]. Synta by itself induced degranulation of mast cells which was not due to cell toxicity. We speculate that this could be due to activation of CRAC channels or a compensatory stimulation of other calcium channels as a result of CRAC blockage. Taken together, our calcium studies suggest that AgNP exposure activates signal transduction pathway(s) that subsequently lead to release of calcium from ER stores and partial activation of CRAC channels thereby leading to influx of extracellular calcium and ultimately degranulation of mast cells.

PI3K is a family of kinases that regulate a wide range of biological functions such as cell growth, differentiation, and survival [71]. Activation of mast cells through IgE and non-IgE receptors (e.g. GPCRs) is associated with phosphorylation and activation of PI3K [72]. It was previously demonstrated that mast cell degranulation through FcεR1 is not PI3K-depenedent albeit PI3K inhibitors (e.g. wortmannin) were shown to decrease degranulation of mast cell in response to FcεR1 activation [73, 74]. Our data show that mast cells exposed to AgNPs had a higher phosphorylation of PI3K and inhibition of PI3K resulted in a significant and an almost complete inhibition of mast cell degranulation in response to AgNP exposure suggesting that PI3K is key component of AgNP-directed mast cells degranulation, unlike the IgE-mediated pathway. This supports our previous data that a potentially different mechanism is regulating mast cell activation in response to AgNPs compared to the IgE-mediated pathway. An important downstream signaling molecule to PI3K and a regulator of the calcium signal in mast cell is PLCγ [31]. In contrast to a previous report where silver ion-mediated increase in calcium was independent of tyrosine kinases and PLCγ [75], our data show that PLCγ is involved in mast cell degranulation in response to AgNP exposure suggesting a potential effect due to the particles (compared to Ag^+^) highlighting a possible different mechanism of mast cell activation. PDZK1 is an intracellular adaptor protein that regulates the expression level and function of SR-B1 [76]. Targeted disruption of the PDZK1 gene resulted in 95% decrease of hepatic SR-B1 expression and was associated with an increase in plasma cholesterol levels [77] suggesting the crucial role of this adaptor protein in regulating the function of SR-B1. It was previously reported that HDL-mediated signaling through SR-B1 involves activation of c-Src, a tyrosine-kinase, and its downstream signaling (including PI3K) which was PDZK1-dependent [78]. Our previous results show that imatinib, a tyrosine-kinase inhibitor, inhibited mast cell degranulation dose-dependently in response to AgNP exposure suggesting that AgNP-mediated degranulation of mast cells involves activation of tyrosine kinases [12]. Accordingly, it is plausible that AgNP-mediated interaction with SR-B1 involves phosphorylation and activation of the tyrosine kinase c-Src and its downstream signaling including phosphorylation of PI3K. Taken together, we postulate that activation of PI3K in response to AgNP exposure might regulate a number of signaling pathways (including PLCγ-IP3-CRAC pathway) that subsequently regulate the calcium signal and ultimately result in mast cell degranulation.

For a better understanding of AgNP-mediated activation of mast cell intracellular signaling pathways, particularly after we demonstrated that AgNP-mediated mast cell degranulation involves activation of PI3K and PLC, we sought to assess other signaling pathways that were previously shown to be involved in mast cell degranulation in response to FcεR1 and non-FcεR1 stimuli [31]. PKC is activated downstream to PI3K and PLCγ activation and was shown to contribute to mast cell degranulation [31]. In addition, it was previously shown that PKC is activated in response to SR-B1 activation [62], and therefore, it was logical to assess its role in response to AgNP-mediated degranulation. Our findings suggest that PKC is involved in mast cell degranulation in response to AgNPs albeit the PKC inhibitor did not block degranulation completely suggesting that other pathways contribute to AgNP-directed mast cell degranulation independent of PKC. Another important signaling molecule is sphingosine-1-phosphate (S1P). S1P is a lipid-signaling molecule that regulates diverse cellular processes such as proliferation, differentiation and secretion [79]. S1P is produced by the action of sphingosine kinases 1/2 (SphK1/2). In the mast cell, S1P is produced following antigen stimulation (i.e. IgE pathway), which plays an important role in regulating the calcium signal and consequent degranulation of mast cells in response to antigen stimulation [80]. Therefore, we assessed involvement of S1P by employing a selective pharmacological inhibitor of SphK. Our results suggest that S1P is not involved in AgNP-mediated degranulation of mast cells. Taken together, our data suggests that mast cell degranulation in response to AgNPs is mediated through a novel pathway that is FcεR1-independent, however, it involves activation of some common signal transduction pathways (activated in response to FcεR1 activation), which are involved in regulating the calcium signal.

RBL-2H3 is a rat basophilic leukemia cell line which has been extensively utilized in studies involving FcεR1 and its downstream signaling [81]. Mast cells are heterogeneous in nature and they are broadly classified into connective tissue- and mucosal-type mast cells (mainly based on the content of their granular proteases) [82]. RBL-2H3 cells were demonstrated to be homologous to rat mucosal mast cells [83]. BMMCs used in the current studies are of the mucosal type (i.e. cultured in the presence of IL-3). We sought to confirm key experiments in RBL-2H3 cells as another model of mucosal-type mast cells. RBL-2H3 cells express SR-B1 (S3 Fig), which does not appear to change in expression following 24 h of AgNPs exposure. RBL-2H3 cells degranulated in response to AgNPs although to a lower extent as compared to BMMCs, which could be attributed to the expression level of SR-B1 in both cells (S3 Fig). One potential reason could be that because these cells form an attached monolayer in the bottom of flask and thus AgNPs require longer time to interact with the cells. Another explanation could be that BMMCs have more cell surface area exposed to AgNPs, that is, BMMCs are in suspension versus RBL-2H3 with only one side (top) interaction. Therefore, we utilized a higher concentration of AgNPs (i.e. 50 μg/ml) to detect a reasonable amount of degranulation that had no major toxicity. Blt-2 and the previously used inhibitors showed a similar trend of inhibition in the RBL-2H3 cells. Similar to BMMCs, AgNPs induced a robust time-dependent phosphorylation of PI3K and PLCγ in RBL-2H3 cells confirming activation of signal transduction pathways in response to AgNP exposure. Overall, our results indicate that RBL-2H3 cells, despite being a different species, respond to AgNPs through activation of potentially the same signaling pathways seen in BMMCs. We anticipate that RBL-2H3 cells may represent a good model to screen a large number of ENMs, by utilizing high-throughput screening techniques, to predict potential degranulation and activation of primary mast cells due to the following reasons: (1) being attached cells; (3) low cost; and (4) ease of use as compared to primary mast cells.

**Fig 7:**
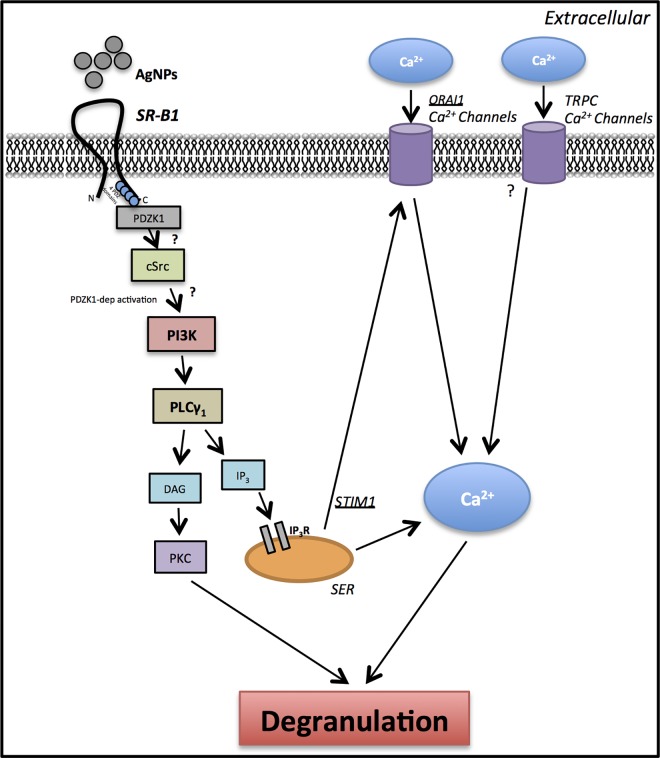
A schematic representation of proposed signaling pathway involved in activation of mast cells by AgNPs We propose that AgNPs interact with SR-B1 leading to recruitment of PDZK1 (SR-B1 adaptor protein), which activates downstream signaling cascade involving PI3K and PLCγ. Inositol 1,4,5-triphosphate (IP_3_), which is released following activation of PLCγ, interacts with its receptor IP_3_R on smooth endoplasmic reticulum (SER) leading to the release of Ca^2+^ from ER stores. As a result of a drop in Ca^2+^ levels in SER, the CRAC Ca^2+^ channels (cell membrane) are activated leading to influx of extracellular Ca^2+^. Increasing intracellular Ca^2+^ levels ([Ca^2+^]_i_) is ultimately culminated in mast cell degranulation. PKC: Protein kinase C; IP_3_ (InsP_3_): inositol triphosphate; PtdIns(4,5)P_2_: phosphatidylinositol-4,5-bisphosphate (PIP_2_); PtdIns(3,4,5)P_3_: phosphatidylinositol-3,4,5-Triphosphate (PIP_3_); PIP_2_ and PIP_3_ are membrane phospholipids; DAG: diacylglycerol. cSrc: cellular sarcoma (protein tyrosine kinase); TRPC: transient receptor potential cation channels.

Overall, our findings revealed that AgNP-mediated degranulation of mast cells involves interaction with SR-B1 and activation of signaling pathways that are involved in regulation of the calcium signal. We demonstrated that ENM-mediated interaction with cells is not only mediated through passive (or even active) uptake by cells but rather a potential direct interaction with cell surface receptors. Based on our findings, we propose that AgNPs interact with SR-B1 thereby activating signal transduction pathways that involve PI3K and PLCγ (possibly downstream to c-Src), which culminates in influx of calcium and degranulation of mast cells (Fig 7). Our current studies suggest that the robust degranulation of mast cells in response to AgNPs exposure is mediated through a novel pathway (potentially through SR-B1); however, it involves activation of common pathways that regulate the calcium signal in response to IgE- and non-IgE stimuli. The data presented in this study provide new insights into a potential mechanism of ENM-mediated activation of mast cells, through interaction with a cell surface receptor and activation of downstream signaling pathways, which could be utilized for potential therapeutic intervention and designing novel ENMs that lack such activation of mast cells.

## Supporting Information

S1 Fig Evaluation of necrotic/apoptotic cell death in BMMCs following AgNPs exposure Cells were treated with AgNPs (25 μg/ml) for 1, 6, and 24 h and necrotic/apoptotic cell death was assessed by staining with propidium iodide (PI) for necrotic cell death and Cy^TM^5 annexin V for apoptotic cell death. (A) Representative graphs of PI/Cy^TM^5 annexin V double stained cells of at least 3 independent experiments. (B) Quantification (average of at least 3 independent experiments) of PI/Cy^TM^5 annexin V double stained cells.
(TIFF)

S2 Fig Evaluation of cell viability in BMMCs and RBL-2H3 following Blt-2 treatment Cells were treated with Blt-2 (50 μM) for 1.5 h and cell viability was assessed. (A) Quantification of PI/Annexin V double stained BMMCs. (B) RBL-2H3 cell viability was assessed by measuring the conversion of MTS into formazan. Values are expressed as mean ± SEM of at least 3 independent experiments.
(TIFF)

S3 Fig Expression of SR-B1 in BMMCs and RBL-2H3 cells Representative immunoblot for the expression of SR-B1 (80 kDa) in BMMC and RBL-2H3 cells in the presence and absence of AgNP (25 μg/ml) for 24 h.
(TIFF)
