# Supplementary figures and images for: Silver Nanoparticle-Directed Mast Cell Degranulation Is Mediated through Calcium and PI3K Signaling Independent of the High Affinity IgE Receptor
Source: PLoS One. 2016 Dec 1;11(12):e0167366. doi: 10.1371/journal.pone.0167366 (PMC5131952; doi:10.1371/journal.pone.0167366)

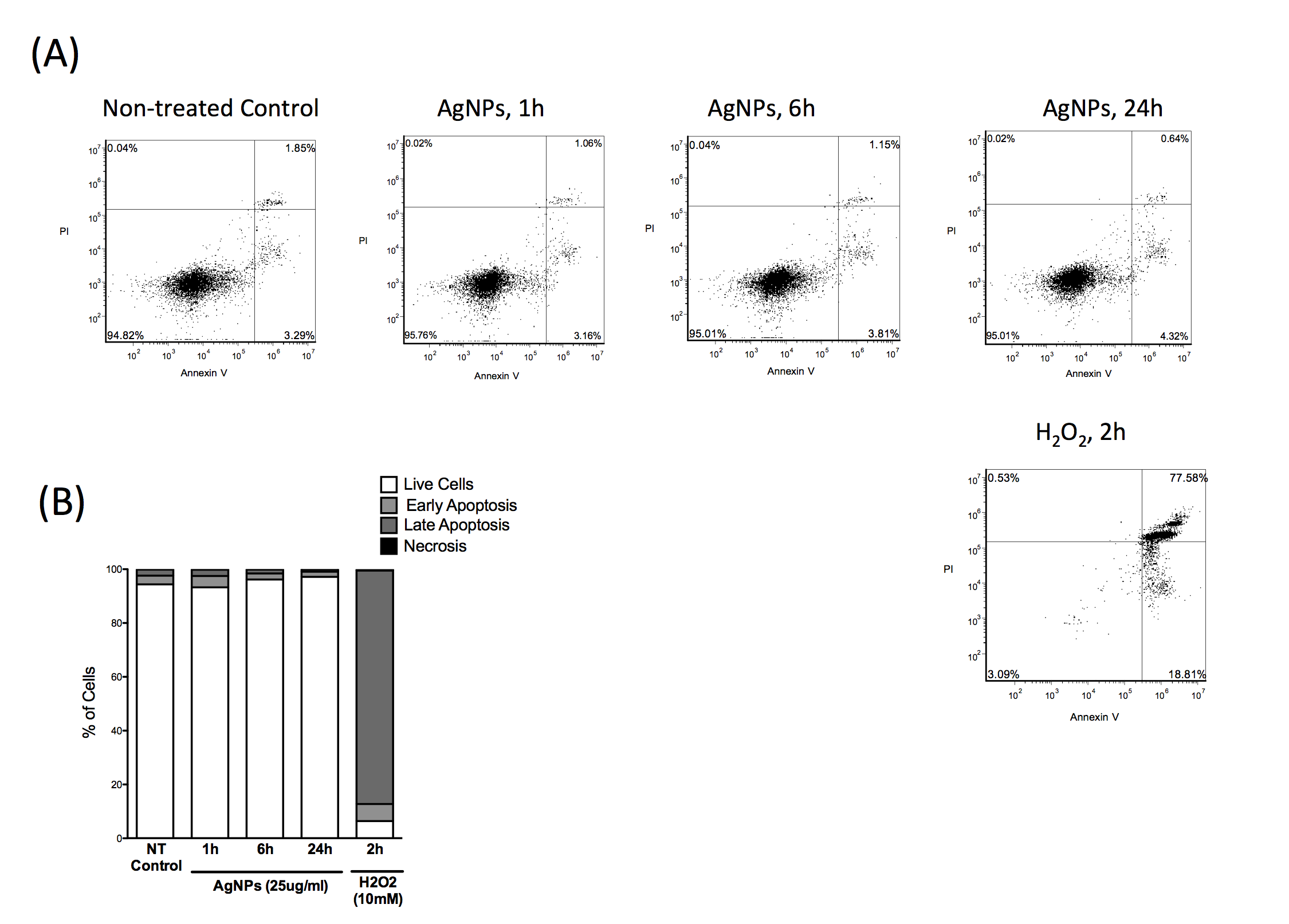

Supplement: S1 Fig — Cells were treated with AgNPs (25 μg/ml) for 1, 6, and 24 h and necrotic/apoptotic cell death was assessed by staining with propidium iodide (PI) for necrotic cell death and CyTM5 annexin V for apoptotic cell death. (A) Representative graphs of PI/CyTM5 annexin V double stained cells of at least 3 independent experiments. (B) Quantification (average of at least 3 independent experiments) of PI/CyTM5 annexin V double stained cells. (TIFF) [file pone.0167366.s001.tiff]

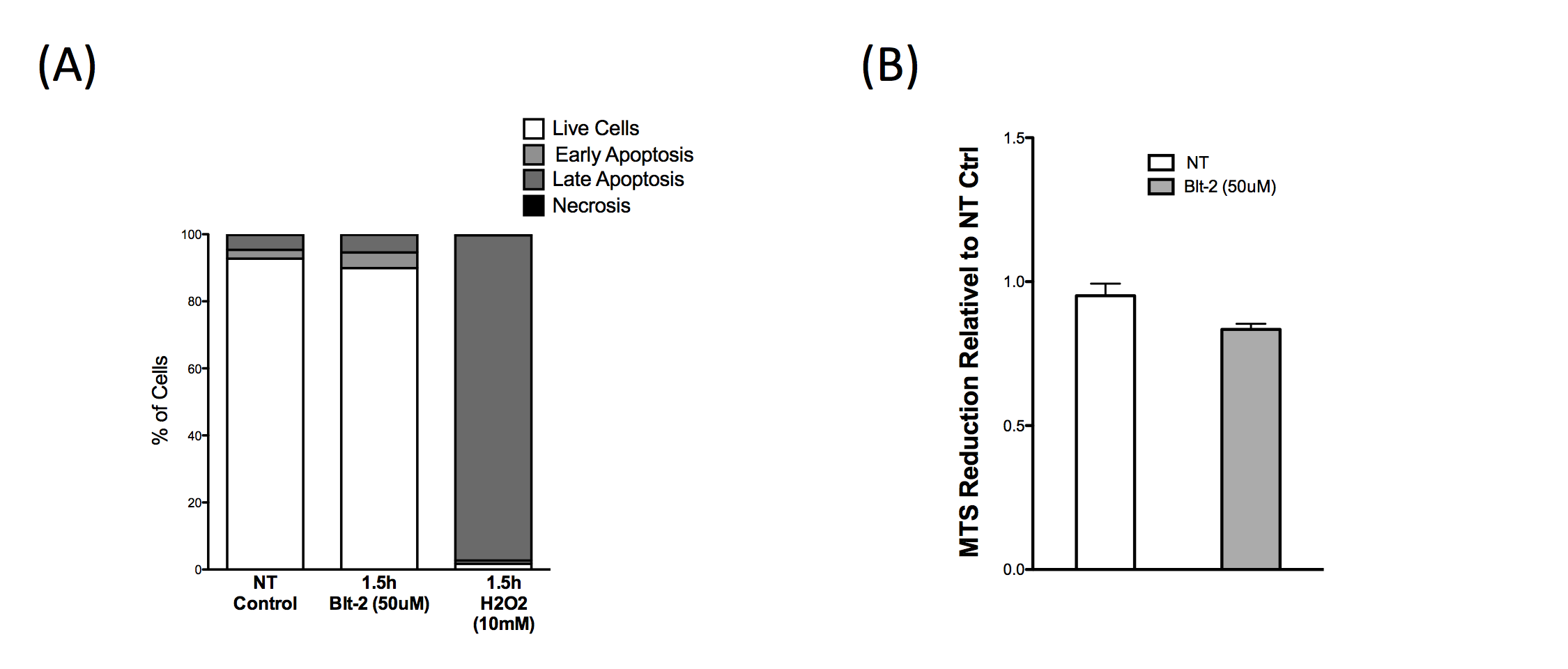

Supplement: S2 Fig — Cells were treated with Blt-2 (50 μM) for 1.5 h and cell viability was assessed. (A) Quantification of PI/Annexin V double stained BMMCs. (B) RBL-2H3 cell viability was assessed by measuring the conversion of MTS into formazan. Values are expressed as mean ± SEM of at least 3 independent experiments. (TIFF) [file pone.0167366.s002.tiff]

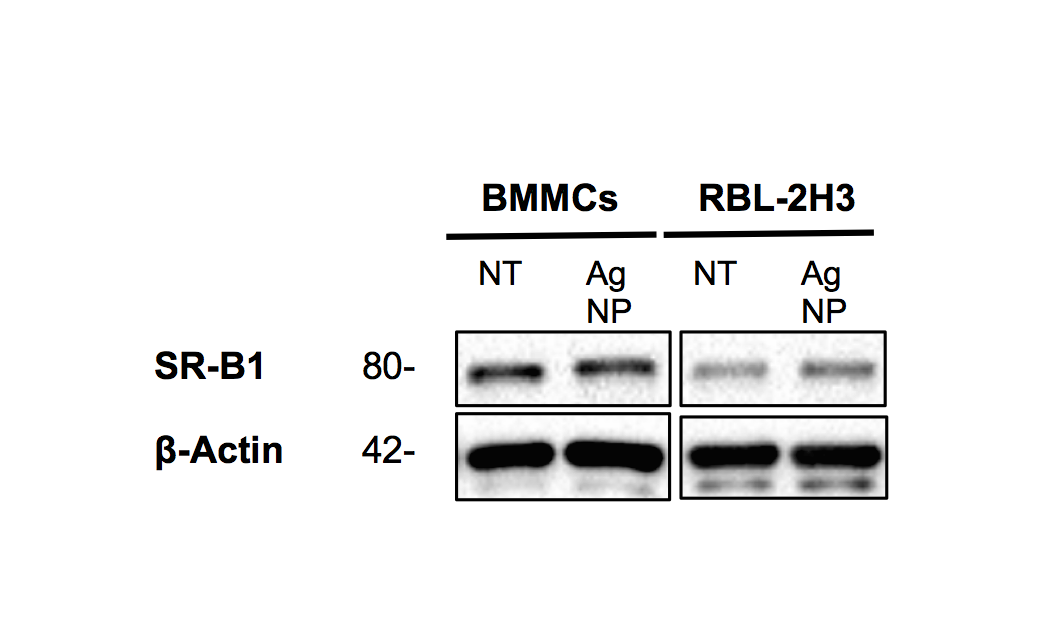

Supplement: S3 Fig — Representative immunoblot for the expression of SR-B1 (80 kDa) in BMMC and RBL-2H3 cells in the presence and absence of AgNP (25 μg/ml) for 24 h. (TIFF) [file pone.0167366.s003.tiff]
